# Time to recover from shock is determinant of a positive fluid balance in septic shock

**DOI:** 10.1186/cc12176

**Published:** 2013-03-19

**Authors:** S Lobo, AL Cunha

**Affiliations:** 1 Faculdade de Medicina de São José do Rio Preto, Brazil

## Introduction

Excess fluids may be harmful in critically ill patients. We aimed to evaluate the cumulative fluid balance during 7 days in patients with septic shock after recovery from shock.

## Methods

A prospective and observational study in septic shock patients. Patients with MAP >65 mmHg and lactate <2.0 mEq/l were included <12 hours after weaning from vasopressors. Daily fluid balance was registered during 7 days after the enrollment. Patients were divided into two groups according to the full cohort's median cumulative fluid balance administered during the period of shock (use of vasopressors) calculated on study day 1: Group 1 ≤4.4 l (*n = *20) and Group 2 >4.4 l (*n = *20).

## Results

**Figure 1:**
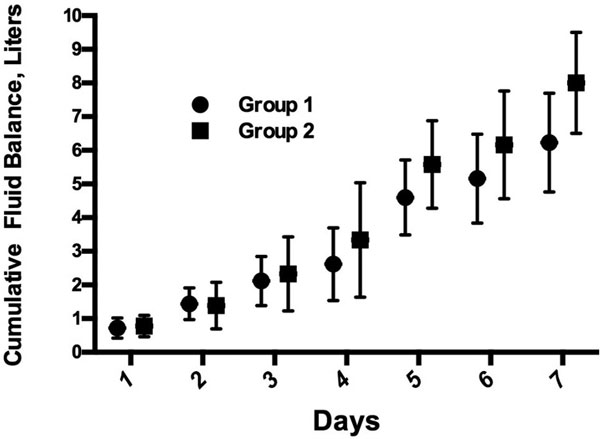


Cumulative fluid balance was 1.6 ± 1.8 l in Group 1 and 10.2 ± 4.1 l in Group 2 and 8.5 ± 5.3 l in Group 1 and 18.5 ± 7.9 l in Group 2 on study day 8 (*P <*0.001 for both). Time for recovery from shock was predictive of receiving larger volume of fluids (OR: 1.38, 95% CI: 1.08 to 1.75, *P *= 0.009). After zeroing fluid balance on study day 2, 7 days cumulative fluid balance continues to increase in both groups (Figure 1). Patients in Group 2 had more prolonged length of stay in the ICU and in hospital than patients in Group 1.

## Conclusion

After recovery from septic shock we notice a huge accumulated fluid balance. A more positive fluid balance was associated with a more prolonged length of stay in the ICU and in the hospital.

